# Shoulder Osteoarthritis

**DOI:** 10.1155/2013/370231

**Published:** 2013-01-10

**Authors:** Claudio Chillemi, Vincenzo Franceschini

**Affiliations:** ^1^Department of Orthopaedics and Traumatology, Istituto Chirurgico Ortopedico Traumatologico (ICOT), via Faggiana 1668, 04100 Latina, Italy; ^2^Department of Orthopaedics and Traumatology, Sapienza University of Rome, ICOT, via Faggiana 1668, 04100 Latina, Italy

## Abstract

Osteoarthritis (OA) is the most frequent cause of disability in the USA, affecting up to 32.8% of patients over the age of sixty. Treatment of shoulder OA is often controversial and includes both nonoperative and surgical modalities. Nonoperative modalities should be utilized before operative treatment is considered, particularly for patients with mild-to-moderate OA or when pain and functional limitations are modest despite more advanced radiographic changes. If conservative options fail, surgical treatment should be considered. Although different surgical procedures are available, as in other joints affected by severe OA, the most effective treatment is joint arthroplasty. The aim of this work is to give an overview of the currently available treatments of shoulder OA.

## 1. Background

Osteoarthritis (OA) is the most frequent cause of disability in the USA [1]. Although not as prevalent as OA of the hip or knee, OA of the shoulder has been demonstrated, in cadaver and radiographic studies, to affect up to 32.8% of patients over the age of sixty years [2, 3] and to be equally debilitating [4]. Patients perceive that the impact of shoulder OA is comparable with that of chronic medical conditions such as congestive heart failure, diabetes, and acute myocardial infarction [5]. The prevalence of shoulder OA increases with age and women appear to be more susceptible than men [6]. 

**Figure 1:**
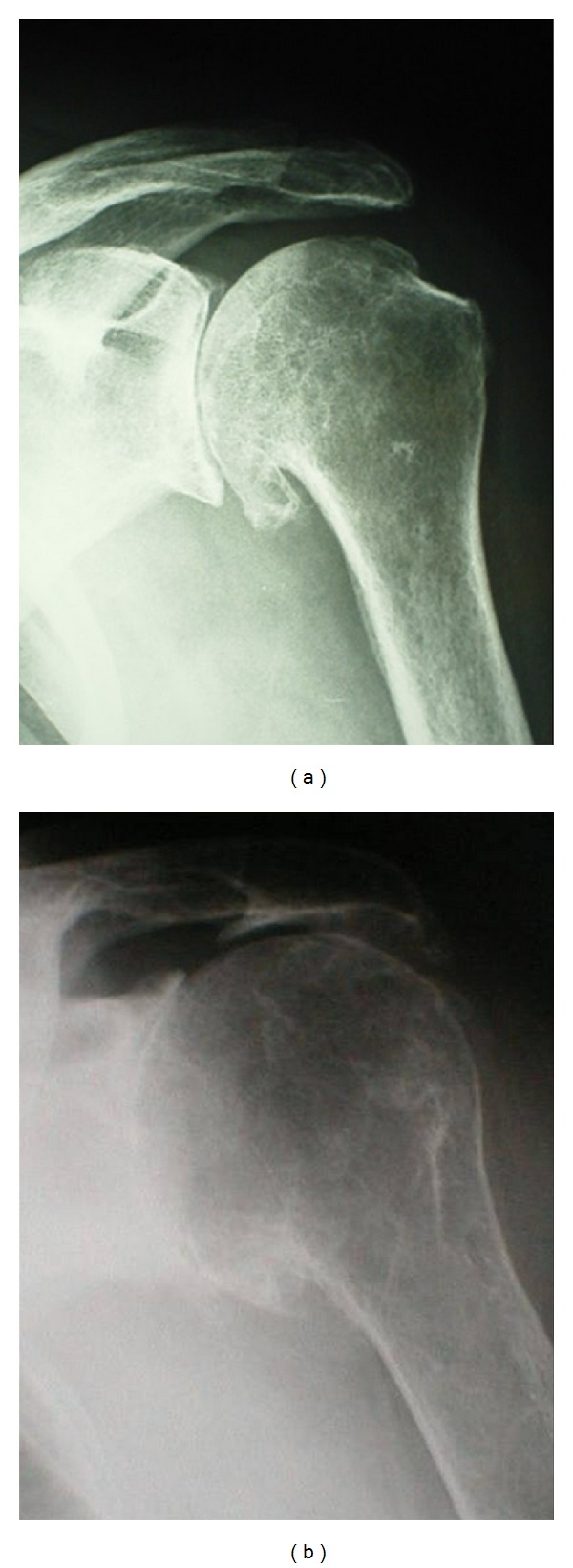
Shoulder OA: radiographic appearance with (a) and without (b) an intact rotator cuff.

OA of the shoulder is the consequence of destruction of the articular surface of the humeral head and glenoid and results in pain and loss of function. It can be primary or secondary. Primary OA is diagnosed when no predisposing factors that could lead to joint malfunction are present. Secondary OA may occur as a result of chronic dislocations and recurrent instability, trauma, surgery, avascular necrosis, inflammatory arthropathy, and massive rotator cuff tears [7, 8] (Figure 1).

Treatment of shoulder OA is often controversial and is typically based on the patient's age, severity of symptoms, level of activity, radiographic findings, and medical comorbidities [9].

Nonoperative treatment options include activity modification, physical therapy, anti-inflammatory drugs (NSAIDs), and intra-articular injections. If conservative options fail, surgical treatment should be considered. Although different surgical procedures are available, as in other joints affected by severe OA, the most effective treatment is joint arthroplasty [10].

The aim of this work is to give an overview of the currently available treatments of shoulder OA.

## 2. Nonsurgical Treatments

Nonoperative modalities should be utilized before operative treatment is considered, particularly for patients with mild-to-moderate OA or when pain and functional limitations are modest despite more advanced radiographic changes [11].

Although nonsurgical management of shoulder OA will not ultimately alter the progression of disease, it can be effective in reducing pain and improve the range of motion [9]. 

Lifestyle modifications and occupational changes are often an initial step in this process. 

Nearly all patients with shoulder OA can benefit from physical therapy. Ideally, therapy should be initiated before the development of atrophy or contracture, and it should be tailored to the specific needs of the patient [8]. Typical programs include gentle range of motion and isometric strengthening of the rotator cuff and scapulothoracic musculature [12]. 

Intra-articular injections are commonly used for patients with OA in other joints and may provide pain relief in patients with shoulder OA [13]. Because of the lack of evidence supporting their efficacy, however, no more than three corticosteroid injections in a single joint are recommendable unless there are special circumstances [11]. Some evidence exists supporting viscosupplementation for shoulder OA. Silverstein et al. [14] reported that glenohumeral viscosupplementation resulted in a significant improvement in shoulder pain and function outcome scores 6 months following injection.

 Medical management of shoulder OA includes salicylates, acetaminophen, and nonsteroidal anti-inflammatory drugs (NSAIDs), which can all be effective in relief of pain and inflammation. In particular randomized trials indicate that NSAIDs are more effective than both paracetamol and placebo for pain relief of OA [15, 16]. It is important, however, to be aware of the increased risk of gastrointestinal and cardiovascular side effects when considering NSAIDs prescription for this cause [16].

## 3. Surgical Treatments

The primary reason to consider surgery for OA is pain that does not respond to nonsurgical measures. Improved function is typically a secondary goal of surgery and is less predictably achieved than pain relief [17]. The choice of treatment then depends on both patient and disease features. Patient features include age, occupation, activity level, and the expectations for functional recovery. Disease features include the lesion size and the extent of chondral involvement.

### 3.1. Arthroscopic Treatment

**Figure 2:**
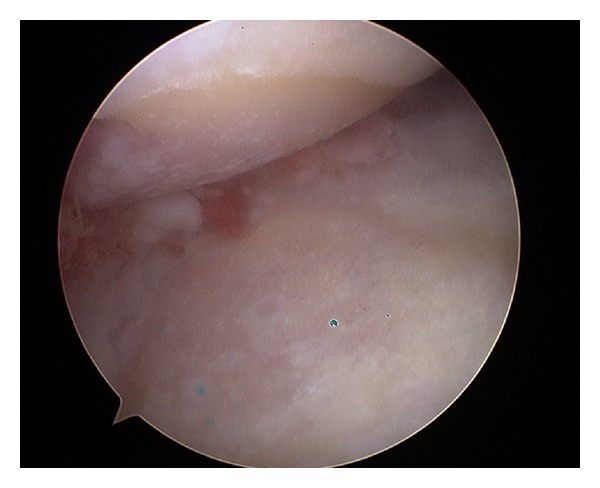
Shoulder OA: arthroscopic view.

Arthroscopy has become increasingly accepted as an option in the management of shoulder OA (Figure 2), thanks to the few complications and low morbidity associated with this procedure [18, 19]. It may be useful both as a diagnostic tool for characterizing lesions and as a therapeutic tool for debridement. Capsular release followed by manipulation may also be an important part of the procedure and can improve postoperative motion [20, 21]. In general, arthroscopic debridement is most likely to benefit patients with mild OA. Although arthroscopic intervention is not likely to halt arthritic progression, it may provide a period of improved pain and function, thereby delaying a larger operation [9]. By stabilizing cartilage lesions, eliminating mechanical symptoms, and releasing capsular contractures, satisfactory outcomes are obtained as reported by several authors [20, 22, 23]. Weinstein et al. [23] described good results from arthroscopic debridement in patients with mild or minimal arthritic change and less favorable results in patients with more advanced changes. Cameron et al. [20] evaluated arthroscopic debridement in patients with grade IV osteochondral lesions, finding an overall 88% rate of postoperative improvement. More recently, Van Thiel et al. [22] described a significant decrease in pain in 55 of 71 patients, mean age 47 years old (range 18–77), after arthroscopic shoulder debridement at a mean of 27 months postoperatively.

### 3.2. Arthroplasty

#### 3.2.1. Humeral Head Resurfacing Arthroplasty

**Figure 3:**
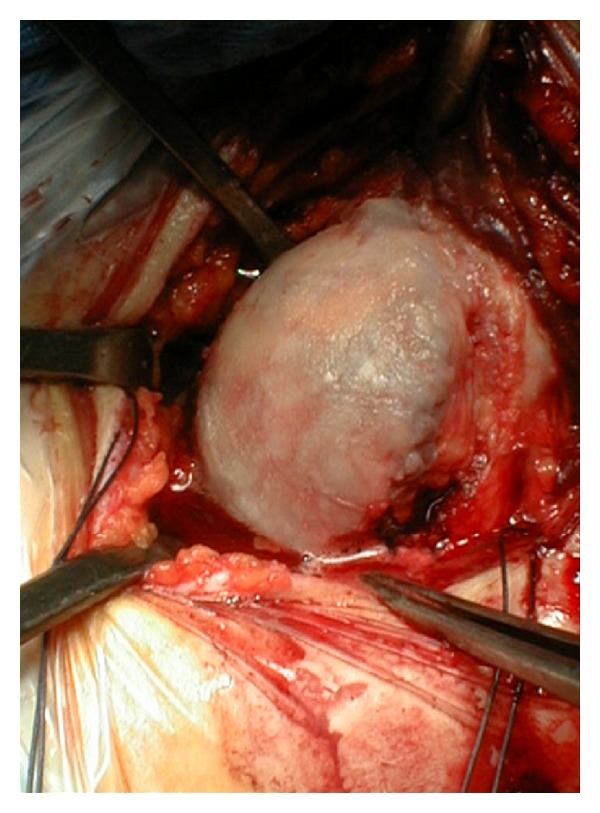
Shoulder OA: intraoperative view of the humeral head.

**Figure 4:**
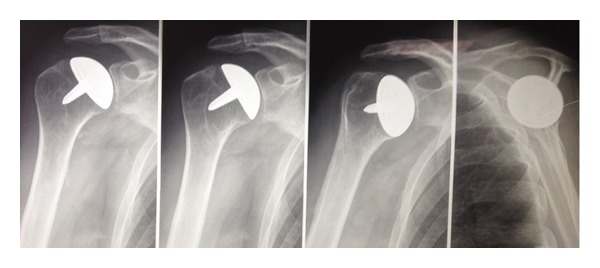
Humeral head resurfacing.

Shoulder resurfacing arthroplasty has gained popularity as an alternative to conventional shoulder arthroplasty for the treatment of OA (Figure 3). In contrast to conventional shoulder arthroplasty, which involves removal of the entire humeral head followed by placement of an intramedullary stem into the proximal aspect of the humerus, shoulder resurfacing consists of reaming the proximal portion of the humeral head and fitting a metal-alloy cap over the remainder of the head [24] (Figure 4). This cap may or may not be mated against a glenoid component [25, 26]. 

Potential advantages of humeral resurfacing are decreased bone resection, shorter operative times, a lower prevalence of humeral periprosthetic fractures, and the potential for straightforward revision to a conventional total shoulder replacement [27, 28]. In addition, it may be straightforward to restore normal offset, inclination, and version of the glenohumeral joint because no osteotomy of the neck is performed and the head-neck angle remains intact [24]. Although many studies demonstrated that the success rates of shoulder surface replacement arthroplasty are comparable with those associated with conventional stemmed prostheses at the time of short and mid-term followup [25, 28, 29], there is lack of evidence regarding long term outcomes and no comparative studies are present. As the bone stock is preserved, resurfacing arthroplasty is particularly indicated in young patients who may require revision to a total shoulder arthroplasty with a stemmed prosthesis during his lifetime. Moreover periprosthetic fractures, which are a concern in this more active population, are less likely to occur than they are with total shoulder replacement because the stem does not pass through the surgical neck [24].

#### 3.2.2. Hemiarthroplasty

**Figure 5:**
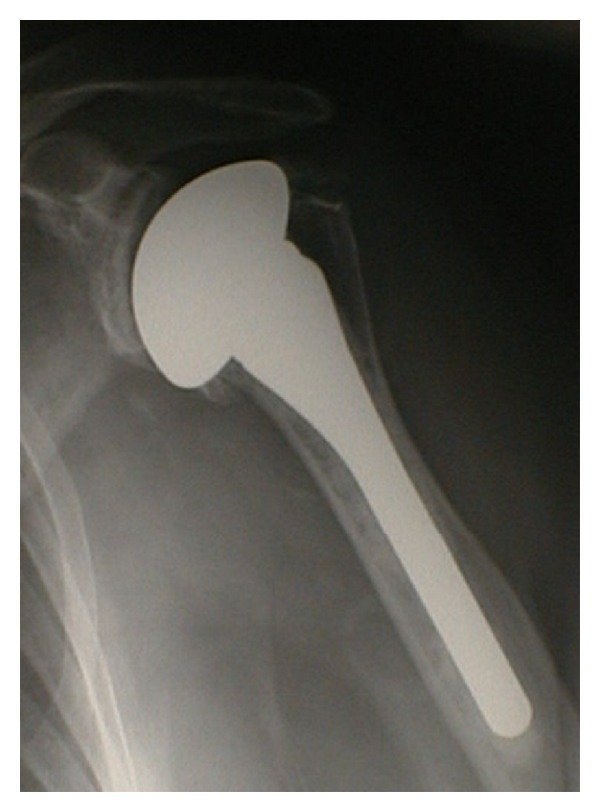
Shoulder hemiarthroplasty.

Both total shoulder arthroplasty and hemiarthroplasty (Figure 5) may achieve good short-term and mid-term results [30–33]. However, while total shoulder arthroplasty may provide superior and more reproducible pain relief, this must be balanced against the technical difficulties of inserting a glenoid prosthesis and the long-term durability of glenoid prostheses in terms of loosening and wear [34–36]. Alternatively, despite good early and mid-term results with hemiarthroplasty, glenoid arthrosis and the need for revision to total shoulder arthroplasty have been demonstrated after longer-term followup [37, 38]. The condition of the glenoid is critical in determining whether humeral head replacement alone will be successful. In particular, patients with concentric glenoid wear and primary OA seems to have better outcomes than those with eccentric glenoid wear and secondary OA [39]. The results of hemiarthroplasty in young individuals appear to deteriorate with time, and there remains a high rate of patient dissatisfaction and revision surgery [40, 41]. 

Sperling et al. [41] found that in spite of long-term improvements in pain relief and function after hemiarthroplasty, in patients under 50 years there was a 60% rate of unsatisfactory results. Several other studies have confirmed that long-term functional results appear to be compromised by progressive glenoid wear, especially in those individuals with preexisting asymmetric glenoid erosion [42]. Thus, primary hemiarthroplasty may be indicated in particular in carefully selected patients with a congruent and minimally arthritic glenoid.

#### 3.2.3. Anatomic Total Shoulder Arthroplasty

**Figure 6:**
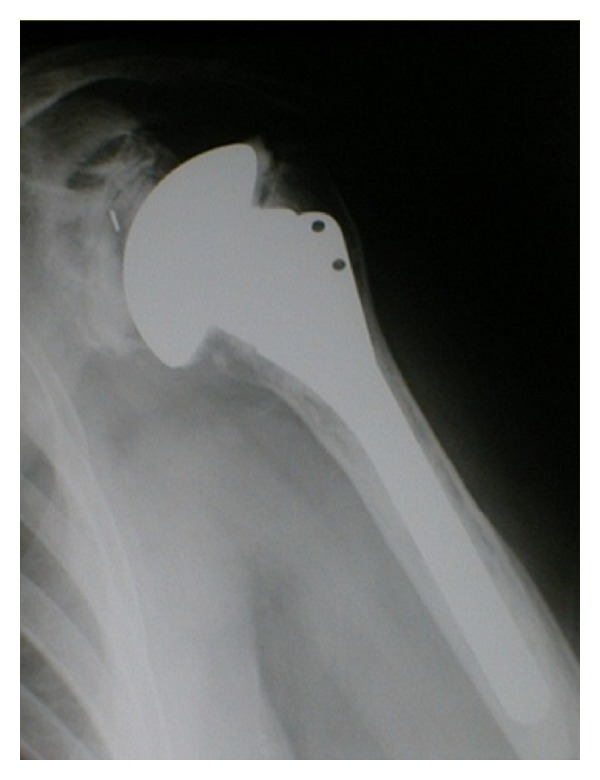
Total shoulder arthroplasty.

Total shoulder arthroplasty (Figure 6) with replacement of the glenoid with a prosthetic polyethylene component is actually the gold standard for the management of advanced and bipolar shoulder OA [31]. Several authors have reported that the functional results of total shoulder arthroplasty are better than those of hemiarthroplasty alone in the treatment of shoulder OA [36, 43]. 

Even in patients under the age of 50 years, survival rates of 97% and 84% at 10 and 20 years have been reported [41]. In a trial of forty-seven patients with primary OA who had been randomized to be treated with total shoulder arthroplasty or hemiarthroplasty and followed for an average of thirty-five months, Gartsman et al. [36] reported significantly greater pain relief (*P* = 0.002) and shoulder motion (*P* = 0.003) after total shoulder arthroplasty. 

In a multicenter nonrandomized study of nearly 700 arthroplasty performed for the treatment of primary arthritis, total shoulder arthroplasty resulted in higher adjusted Constant scores (96% versus 86% after hemiarthroplasty) and improved motion (forward elevation, 145° versus 130° after hemiarthroplasty, and external rotation, 42° versus 36° after hemiarthroplasty [43]). Finally, a 2005 meta-analysis of 112 patients demonstrated that total shoulder arthroplasty resulted in higher functional outcome scores, greater pain relief, and increased shoulder motion at two years postoperatively [44]. However, these benefits come with the risk of glenoid loosening [45]. Particularly in younger, more active patients, long-term survival of the glenoid component is a concern because the outcomes of glenoid revision are not as robust as the outcomes of primary total shoulder arthroplasty [46]. In a recent review of 33 previously published studies, Bohsali et al. [47] found that glenoid component loosening accounted for 39% of all complications after total shoulder arthroplasty. Sperling et al. [41] similarly reported high rates of loosening and declining prosthesis survival after 5 to 8 years, specifically in younger individuals. Soft-tissue failure and prosthetic instability may explain, in part, the high rate of glenoid loosening [48]. In addition, the risk of glenoid failure seems to be associated with the use of reaming to optimize the seating and positioning of the glenoid component. The reaming of the glenoid surface weakens the support from subchondral bone exposing the component to excessive compressive and eccentric forces. Preserving subchondral bone may then be important for long-term longevity of the glenoid component [49].

Given the risk of glenoid loosening, careful patient selection for total shoulder arthroplasty is paramount. It is a durable and effective option in appropriately selected and counseled individuals who have had failure with all palliative and reconstructive treatment modalities [8].

#### 3.2.4. Reverse Total Shoulder Arthroplasty

While anatomic total shoulder arthroplasty can be considered a very effective treatment for shoulder OA in the presence of an intact rotator cuff, when shoulder OA is associated with a massive rotator cuff rupture (i.e., cuff tear arthropathy-CTA [50]), the results are suboptimal. The rotator cuff is an active stabilizer that is indispensable for the proper functioning of the glenohumeral joint [51]. With a massive rupture, the center of rotation of the joint migrates upward and joint stresses become off-center, which may explain the glenoid loosening observed with total shoulder prostheses [52]. To avoid this problem, it is possible to leave the glenoid in place and to carry out only a hemiarthroplasty but the results are often somewhat disappointing and the improvement in shoulder function and range of motion is limited [53, 54]. Moreover the progressive upward displacement of the humeral head causes wear of the coracoacromial arch and the patient is at risk for a deteriorating functional result over time [55].

**Figure 7:**
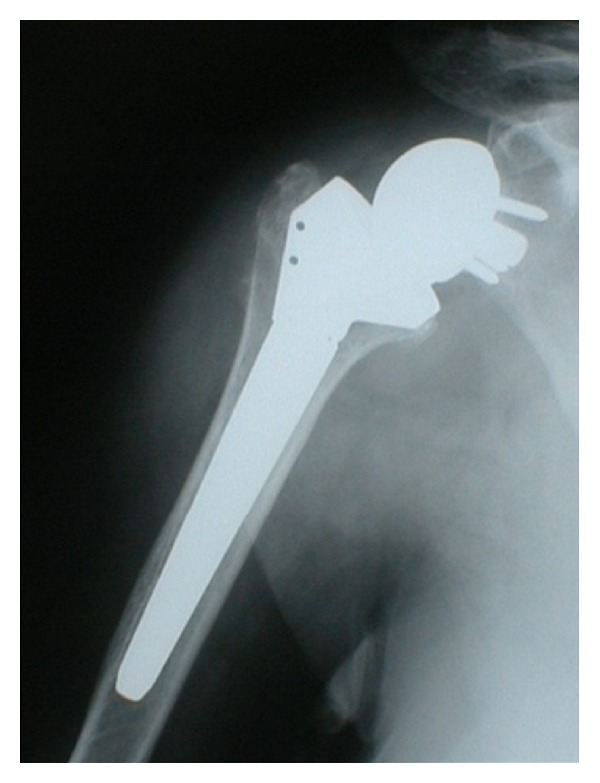
Reverse shoulder prosthesis.

Reverse total prostheses (Figure 7) such as those developed by Grammont et al. [56] appear to provide good functional results in CTA [57, 58]. 

The congruent joint surfaces of the reverse ball-and-socket design provide inherent stability, while moving the joint center of rotation medially and distally to increase deltoid function and the range of motion [59, 60]. Key aspects of the modern reverse total shoulder arthroplasty include (1) a large glenosphere component with no neck, which allows medialization of the center of rotation and reduced torque on the glenoid component; (2) a humeral implant with a nonanatomic valgus angle, which moves the center of joint rotation distally, thus maximizing the length and tension of the deltoid to increase its ability to abduct the humerus, in addition to providing increased stability; and (3) a greater range of shoulder motion [61]. Distal displacement of the center of joint rotation increases the lever arm of the deltoid and also recruits portions of the anterior and posterior heads of the deltoid to act as abductors of the arm, permitting elevation above shoulder height. In addition, reestablishment of the subacromial space permits greater potential abduction [61, 62]. 

Reverse total shoulder arthroplasty has been shown to be effective in treating CTA, with numerous studies demonstrating improvements in shoulder motion and patient outcome [56–59]. However, most reports have presented only midterm followup results, and despite these encouraging midterm results, complications have been reported. In one long-term analysis, Molé and Favard reported the radiographic appearance of deterioration after approximately five to six years, with clinical deterioration appearing after approximately eight years [63]. In a retrospective review of eighty reverse total shoulder arthroplasties, with a mean duration of followup of forty-four months and a mean patient age of 72.8 years, Sirveaux et al. [57] reported an increase in the mean Constant score from 22.6 points preoperatively to 65.6 points postoperatively, with 96% of the patients having little or no pain and an increase in mean active forward flexion from 73° to 138°. However, at the time of followup, 4% of the implants had failed and been revised, 6% were noted to have radiographic signs of loosening, and 9% demonstrated unscrewing of the glenosphere component. In contrast, Guery et al. [51] showed that the global survivorship of the Grammont reverse total shoulder prosthesis with revision or loosening as the end point is good even eight years after implantation. Moreover, Cuff et al. [64] recently reported durable clinical and radiographic results and a survival rate of 94% at 5 years of followup. In addition, no mechanical baseplate failures or glenoid-sided screw loosening was noted. Thus, although the results of reverse total shoulder arthroplasty are promising with regard to the postoperative range of motion, pain relief, and improvements in clinical outcome, long-term studies are necessary to confirm the encouraging data on survivorship reported in recent works.

## 4. Conclusions

Shoulder OA can be a major source of pain and disability. The management of this condition, in particular in young active patients, is a challenge, and the optimal treatment has yet to be completely established. If nonoperative treatment fails, several surgical techniques are currently available. Shoulder arthroplasty produces excellent and reliable functional improvements, but further studies will be necessary to confirm the long-term effectiveness of this procedure.
